# Influence of personality disorders on sexual behaviours and response to treatment of psychogenic erectile dysfunction in phosphodiesterase 5 inhibitor non-responders

**DOI:** 10.3389/fpsyg.2024.1496891

**Published:** 2024-10-23

**Authors:** Marina Cabello-García, Yolanda Sánchez-Sandoval, Antonio Daniel García-Rojas

**Affiliations:** ^1^Department of Psychology, University of Malaga, Málaga, Spain; ^2^Biomedical Research and Innovation Institute of Cadiz, INIBICA, Cadiz, Spain; ^3^Department of Psychology, University of Cadiz, Cadiz, Spain; ^4^Department of Pedagogy, University of Huelva, Huelva, Spain

**Keywords:** erectile dysfunction, personality disorders, phosphodiesterase-5 inhibitors, psychosexual therapy, couple wellbeing, sexual behaviours

## Abstract

**Background:**

Personality disorders may influence sexual behaviours and sexual dysfunction.

**Aim:**

Our main objective was to analyse the influence of personality disorders (PDs) in patients with erectile dysfunction (ED) of psychological origin that fail to respond to andrological treatment with Phosphodiesterase-5 inhibitors (IPDE5), assessing whether there are differences in sexual behaviours and response to psychosexual treatment.

**Methods:**

The research is designed as an *ex post facto* retrospective study with two groups. A control group of 23 men with ED without personality disorders and a group of 51 men with both ED and PDs.

**Results:**

In the case sample, 34.30% of the participants presented more than one personality disorder. No significant differences were found in sexual behaviours except for heteromasturbation (men without PDs masturbated their partners more to satisfy them than men with PDs), and men with PDs considered themselves less premature ejaculators than the control group. Finally, 82.14% of the control group did well with psychosexual therapy compared to 53.85% of the PDs group.

**Conclusion:**

Psychosexual treatment of ED has a worse outcome if the men also have PDs. Strengths and Limitations: from a clinical standpoint, it is important to assess the presence of personality disorders in men with ED and to implement psychosexual strategies to improve the response to treatment in these cases. Confirmation of the results with a much larger sample becomes necessary.

## 1 Introduction

Erectile dysfunction (ED) is defined as the persistent inability to achieve or maintain an erection sufficient for satisfactory sexual performance (NIH, 1993). According to the Diagnostic and Statistical Manual of Mental Disorders, 5th Edition (DSM-5), erectile disorder of psychogenic origin is diagnosed on the basis of several specific criteria (APA, 2024). These include marked difficulty in achieving or maintaining an erection during sexual activity, or a significant reduction in erectile rigidity, experienced in at least 75–100% of sexual encounters. The symptoms must have persisted for at least 6 months, causing significant distress in the individual, and must not be attributable to external factors such as substance use or medical conditions.

In addition, the DSM-5 describes personality disorders as patterns of behaviour and inner experience that deviate significantly from cultural expectations. These deviations are manifested in at least two of the following domains, including cognition, affect, interpersonal functioning and impulse control. In short, according to the American Psychiatric Association (APA), personality disorders (PDs) are inflexible and maladaptive personality traits that are exhibited in a wide range of personal and interpersonal contexts (APA, 2024).

From an aetiological standpoint, erectile dysfunction is classified as organic, psychological or mixed (NIH, 1993), in such a way that 34.5% of cases are estimated to be organic, 18.1% psychogenic and 47.5% mixed (organic/psychogenic) (Mirone et al., 2005). Despite the high incidence, the psychological factor has not been widely studied (Mirone et al., 2021). ED has been linked to altered intimacy, marital conflict, stress (Lizza and Rosen, 1999), performance anxiety and anxiety disorders (Barlow, 1986; Beck and Barlow, 1986; Cannarella et al., 2021; Lizza and Rosen, 1999; Velurajah et al., 2021), cognitive, affective and emotional aspects (Cranston-Cuebas and Barlow, 1990; Hu et al., 2024; Nobre, 2010; Wiegel et al., 2007), depressive symptoms, low frustration tolerance, guilt and sensitivity to rejection (Derogatis et al., 1981; Derogatis and Meyer, 1979; Yuan et al., 2023), increased hostility and self-esteem (Bancroft et al., 2005; DiMeo, 2006; Özkent et al., 2021). However, there are few studies linking personality disorders to ED. In a recent systematic review on personality disorders and sexual dysfunction (Cabello-García et al., 2020), personality disorders were shown to influence sexual response, but of the few 14 articles that met the review’s inclusion criteria, only one related personality disorders to erectile dysfunction at the clinical level, showing a high level of neuroticism in erectile problems (Quinta Gomes and Nobre, 2011). Subsequent to that review, other research found that personality disorders influenced ED and that narcissistic individuals improved the most with andrological treatment (Ajo et al., 2021).

Despite these findings, the relationship between personality disorders and erectile dysfunction remains an important gap in the scientific literature. Most studies have focused on general emotional and psychological factors, leaving unexplored in depth how different types of personality disorders, beyond narcissism, affect erectile dysfunction. This lack of specific studies in this area underscores the need for further research to better understand the impact of personality disorders on ED.

This study has significant social and academic relevance. From a societal point of view, understanding how personality disorders influence erectile dysfunction may improve the accuracy of diagnoses and therapeutic interventions, allowing for more personalised and effective treatment for individuals with ED. Furthermore, given that ED not only affects sexual health, but may also impact interpersonal relationships and overall quality of life, the findings of this study may contribute to the development of better clinical strategies to improve patients’ psychological and relational well-being.

Academically, this study addresses a critical gap in the literature by exploring the connection between personality disorders and erectile dysfunction, an area that has been under-researched. By identifying specific patterns, this study will bring new insights to clinical psychology and sexology and may lay the groundwork for future research on the relationship between personality disorders and sexual dysfunction.

The present study aims to verify whether there are differences in sexual behaviours and response to psychosexual therapy between men with ED without PDs and men with ED who also have PD criteria and who have not progressed favourably with IPDE5 treatment, following Theodor Millon’s personality assessment model through the Millon Clinical Multiaxial Inventory (MCMI-III) (Millon, 2011; Millon and Grossman, 2005), which differentiates personality disorders into clinical patterns (Schizoid, Phobic, Dependent, Histrionic, Narcissistic, Antisocial, Sadistic Aggressive, Compulsive, Passive-Aggressive, Self-Destructive), severe personality pathology (Schizotypal, Borderline and Paranoid), clinical syndromes (Anxiety, Hysteriform, Hypomania, Depressive Neurosis, Alcohol Abuse, Drug Abuse) and/or severe syndromes (Psychotic Thinking, Major Depression and Psychotic Delusions).

Ultimately, the aim is to find out whether there are differences in the response to treatment between men without PDs and those with PDs, based on the hypothesis that men with ED and PDs have lower adherence to sex therapy and therefore a higher rate of dropout and therefore therapeutic failures than men with ED without PDs.

## 2 Materials and methods

### 2.1 Study design

Following the methodological classification proposed by Montero and León (2007), the present research would fall into the category of a retrospective *ex post facto* study with two groups.

### 2.2 Population

Sampling was non-probability and convenience sampling. This sample consisted of patients who sought psychosexual therapy for erectile dysfunction that had not subsided with the use of phosphodiesterase 5 inhibitors. An interview was conducted to verify whether patients met the inclusion criteria, and did not meet the exclusion criteria. Treatment was carried out on an individual basis. Patients were informed of the therapy to be performed and were asked for their authorisation to take part in the present study, subsequently signing the consent form.

A sample of 51 people aged between 18 and 60 years with psychogenic erectile dysfunction, according to DSM-5 criteria, and with a Golombok Rust Inventory of Sexual Satisfaction (GRISS) (Rust and Golombok, 1986) dysfunctionality score higher than 4.5 (cut-off) on the A scale. This score was validated in Spanish in 2021 (Cabello-Santamaría et al., 2021) and correlates with the sexual function domain of the IIEF (Cabello-Santamaría, 2004). The GRISS questionnaire likewise assesses scales of sensuality, frequency, communication, satisfaction and ejaculation. The sample was split into a case group of 28 people with ED and PDs diagnosed by a score higher than 75 on one of the scales of the Millon Clinical Multiaxial Inventory (MCMI-III) questionnaire and a control group of 23 people with ED without PDs. All participants had a partner of more than 6 months’ standing and had been taking an IPDE5 for at least 4 months without satisfactory results. The groups were balanced by selecting participants with similar characteristics (see Table 1). The exclusion criteria were that they were taking any medication (although the current literature does not link these drugs to ED), that they had a sexual orientation other than heterosexual (the GRISS questionnaire is validated only in a heterosexual population), and that they had not completed the interview and questionnaires used in the sexual research. Briefly, two different groups were compared, one presenting ED with DSM-5 criteria and with a dysfunctional score on the GRISS (above 4.5 on scale A corresponding to ED) and the other with the same criteria for ED and also a score above 75 on one of the scales of the Millon Clinical Multiaxial Inventory (MCMI-III) questionnaire.

**Table 1:** Descriptive data.

	Case group		Control group		*p*
	Mean	Standard deviation	Mean	Standard deviation	
Age	37.86	2.63	37.97	1.56	0.760
Time as couple (months)	87.79	125.38	74.51	96.80	0.804
Number of sexual partners	13.21	21.68	16.23	16.23	0.740
Number of stable partners	8.14	1.78	7.17	1.73	0.172
Foreplay duration (minutes)	18.29	15.08	15.92	14.47	0.482

*p = statistical significance using Mann–Whitney U test.*

The following variables from the clinical history were also assessed (Cabello-Santamaría, 2004, 2010): age, time as a couple; lifetime or acquired sexual dysfunction (primary or secondary); oral sex practise; anal sex practise; use of erotic toys; masturbatory behaviours; individual masturbation and masturbation in a couple; presence of erotic fantasies during sexual intercourse; extra-partner sexual relations; consumption of illegal drugs for sexual activity; subjective quality of the couple’s relationship (ruling out sexual aspects); dropout (therapy failure) or success of therapy.

### 2.3 Questionnaires

Golombok Rust Inventory of Sexual Satisfaction (GRISS) (Cabello-Santamaría, 2004; Cabello-Santamaría et al., 2021). This questionnaire assesses the presence of sexual dysfunctions. It consists of 28 items to be answered on a Likert-type scale with 5 response options. The questionnaire measures the following scales: non-sensuality, dissatisfaction, infrequency, non-communication, avoidance, anorgasmia (women), vaginismus (women), premature ejaculation (men) and erectile dysfunction (men). There is a version for men and another for women. Reliability is adequate in terms of internal consistency and test–retest, as well as adequate convergent/divergent validity and ability to discriminate between patients and control group (Cabello-Santamaría, 2004; Cabello-Santamaría et al., 2021; Golombok et al., 1984). The authors report Cronbach’s alpha reliability values of 0.94 for the male version and 0.87 for the female version.

Millon Clinical Multiaxial Inventory (MCMI-III) (Millon, 2011; Millon and Grossman, 2005). The questionnaire assesses 22 scales grouped into 4 blocks: 10 basic personality scales [Schizoid, Phobic (avoidant), Dependent (submissive), Histrionic, Narcissistic, Antisocial, Aggressive-sadistic, Compulsive, Passive-aggressive, Self-destructive], 3 Pathological Personality scales (Schizotypal, Borderline, Paranoid), 6 Clinical Syndromes of moderate severity (Anxiety, Hysteriform, Hypomania, Depressive Neurosis, Alcohol Abuse, Drug Abuse), and 3 Clinical Syndromes of severe severity (Psychotic Thinking, Major Depression, Psychotic Delusions). The pathological personality scales and clinical syndromes differ from the 10 basic scales on a number of criteria, most notably by their deficits in social competence and frequent psychotic episodes, as they are especially vulnerable to the everyday stresses of life, less integrated in terms of personality organisation and less effective in coping than the 10 milder types.

The Dyadic Adjustment Scale by Spanier, specifically the item that numerically assesses the degree of partner happiness (Spanier, 1976).

### 2.4 Psychosexual therapy

The people in the sample, after having failed treatment with IPDE5, participated in a sex therapy programme divided into five sections. The therapeutic strategy has been applied in the clinical centre attended by the patients since 2004, described in the literature (Cabello-Santamaría, 2004, 2010), and is a compendium of the classic Masters and Johnson therapy (Masters and Johnson, 1970), the therapeutic model of Kaplan (1978), the therapeutic model of Zwang (1985), and more specifically the strategies proposed by Hawton (1992) implemented with mindfulness techniques (Dascalu and Brotto, 2018). Likewise, as the literature points out, special emphasis is placed on the first consultation to facilitate the therapeutic alliance (del Río et al., 2022).

### 2.5 Statistical analysis

Descriptive statistics were used (mean, standard deviation, percentages, etc.). The Kolmogorov–Smirnov test was performed to check the assumption of normality of the sample. The non-parametric Mann–Whitney U test was performed for hypothesis testing, as well as the Chi-Square test using the contingency table.

## 3 Results

First, the Kolmogorov–Smirnov test was performed to verify the normality of the sample, and thus decide the type of contrast test to perform. The results showed that the sample did not meet the normality criterion (*p* = 0.000), so non-parametric contrasts were performed.

The results indicated that the case sample (ED + PDs) and the control sample (ED without PDs) were homogeneous, as shown in Table 1. They did not differ in terms of age, time spent with a partner, number of sexual partners, number of steady partners or time spent in erotic play. In the case group, 76.92% had a dysfunctional score in compulsive personality, 35.90% had a dysfunctional score in dependent personality, and 28.21% had a dysfunctional score in schizoid personality (see Table 2). Moreover, 34.30% had more than one dysfunctional score on the personality variables.

**Table 2:** Personality scale percentages by groups.

Scales		Cases		Control	
		*N*	%	*N*	%
Schizoid	Functional	28	71.79%	28	100%
	Non-functional	11	28.21%	0	0%
Avoidant	Functional	32	82.05%	28	100%
	Non-functional	7	17.95%	0	0%
Dependent	Functional	25	64.10%	28	100%
	Non-functional	14	35.90%	0	0%
Histrionic	Functional	32	82.05%	28	100%
	Non-functional	7	17.95%	0	0%
Narcissist	Functional	31	79.49%	28	100%
	Non-functional	8	20.51%	0	0%
Antisocial	Functional	34	87.18%	28	100%
	Non-functional	5	12.82%	0	0%
Aggressive-Sadistic	Functional	35	89.74%	28	100%
	Non-functional	4	10.26%	0	0%
Compulsive	Functional	9	23.08%	28	100%
	Non-functional	30	76.92%	0	0%
Passive-aggressive	Functional	36	92.31%	28	100%
	Non-functional	3	7.69%	0	0%
Self-destructive	Functional	36	92.31%	28	100%
	Non-functional	3	7.69%	0	0%
Schizotypal	Functional	35	89.74%	28	100%
	Non-functional	4	10.26%	0	0%
Limit	Functional	37	94.87%	28	100%
	Non-functional	2	5.13%	0	0%
Paranoid	Functional	31	79.49%	28	100%
	Non-functional	8	20.51%	0	0%

No differences were found in sexual communication with the partner, in the time dedicated to erotic games and behaviours (“non-sensuality” variable of the GRISS), nor were there differences in sexual frequency or satisfaction, although there were differences in premature ejaculation, which was higher in the case group (see Table 3). Differences were also found in the practise of heteromasturbation (control case partners masturbated together, or to each other, more frequently than PD cases; see Table 4).

**Table 3:** Mean score, standard deviation and contrast of the GRISS questionnaire scales by groups.

Variable	Cases		Control		
	Mean	Standard deviation	Mean	Standard deviation	*p*
Erectile dysfunction	6,90	1,17	7,14	1,48	0,447
Premature ejaculation	4,59	2,24	5,93	2,16	0,026*
Non-sensuality	1,67	1,15	2,04	1,43	0,231
Avoidance	5,00	2,29	4,21	2,25	0,112
Dissatisfaction	4,77	2,02	4,79	1,87	0,995
Non-communication	4,00	1,88	4,07	1,74	0,933
Infrequency	5,74	1,83	5,68	1,76	0,781

* = <0.05; *p* = statistical significance using Mann–Whitney U.

**Table 4:** Frequency, percentage and contrast of behaviours by groups.

		Cases		Control		
		*N*	%	*N*	%	*p*
Sexual dysfunction	Primary	10	25.64%	9	32.14%	0.560
	Secondary	29	74.36%	19	67.86%	
Oral sex	No	7	17.95%	3	10.71%	0.412
	Yes	32	82.05%	25	89.29%	
Anal sex	No	31	79.49%	21	75.00%	0.664
	Yes	8	20.51%	7	25.00%	
Use of sex toys	No	25	64.10%	18	64.29%	0.988
	Yes	14	35.90%	10	35.71%	
Self-stimulation	No	1	2.56%	3	10.71%	0.165
	Yes	38	97.44%	25	89.29%	
Heteromasturbation	No	26	66.67%	9	32.14%	0.005**
	Yes	13	33.33%	19	67.86%	
Erotic fantasies	No	16	41.03%	11	39.29%	0.886
	Yes	23	58.97%	17	60.71%	
Extra-partner sexual relations	No	30	76.92%	26	92.86%	0.082
	Yes	9	23.08%	2	7.14%	
Illegal drug consumption	No	36	92.31%	24	85.71%	0.384
	Yes	3	7.69%	4	14.29%	
Tobacco consumption	No	29	74.36%	19	67.86%	0.560
	Yes	10	25.64%	9	32.14%	
Alcohol consumption	No	11	28.21%	8	28.57%	0.974
	Yes	28	71.79%	20	71.43%	

** = <0.01; *p* = statistical significance using Chi-Square.

The case group’s scores on the avoidant, histrionic, narcissistic, antisocial, aggressive-sadistic, passive-aggressive, self-destructive, borderline, hypomanic, psychotic-delusional and major depression scales did not differ significantly from the control group in terms of improvement in psychosexual therapy. In contrast, there were significant differences in the scores recorded in the schizoid, dependent, schizotypal, paranoid, anxiety, somatoform, neuroticism, tendency to abuse alcohol and drugs, and compulsive scales, all of which responded worse to therapy, with a higher dropout rate (see Table 5).

**Table 5:** Mean score, standard deviation and contrast of the MCMI-II questionnaire scales by groups.

	Cases		Control		
	Mean	Standard deviation	Mean	Standard deviation	*p*
Schizoid	60.26	27.48	40.71	20.04	0.003**
Avoidant	45.49	30.91	40.14	29.81	0.457
Dependent	64.72	27.00	44.64	24.71	0.002**
Histrionic	44.26	28.16	41.18	21.81	0.909
Narcissist	49.21	30.61	40.21	21.05	0.274
Antisocial	37.21	30.38	24.93	19.63	0.201
Aggressive-Sadistic	42.90	27.97	32.57	20.66	0.137
Compulsive	91.15	21.41	64.07	14.20	0.000**
Passive-aggressive	34.44	25.24	24.14	18.98	0.097
Self-destructive	37.56	27.01	29.43	22.69	0.285
Schizotypal	47.05	23.25	34.00	22.38	0.034*
Limit	38.54	21.70	30.32	18.47	0.143
Paranoid	56.51	26.90	40.79	21.28	0.018*
Anxiety	57.08	25.77	38.68	22.99	0.006**
Somatoform	57.13	26.55	33.64	18.05	0.000**
Hypomania	41.49	26.36	36.14	19.64	0.606
Dysthymia	52.77	26.57	38.75	21.57	0.041*
Alcohol abuse	36.05	24.77	24.64	15.87	0.048*
Drug abuse	34.23	24.49	20.29	14.36	0.017*
Psychotic thinking	38.62	23.61	32.96	23.99	0.279
Major depression	43.23	26.37	31.82	27.15	0.094
Psychotic delusions	53.10	25.41	41.43	18.41	0.073

* = <0.05; ** = <0.01; *p* = statistical significance using Mann–Whitney U test.

As for the assessment of the quality of the couple’s relationship, excluding sexuality, men with ED without PDs rated the relationship more positively, with statistical significance. Regarding treatment progress and completion, there was a higher percentage of people who completed the treatment in the control group compared to the case group (82.14% > 53.85%). It can be concluded that ED comorbid with a PD has a worse therapeutic prognosis than ED without PDs.

## 4 Discussion

The results show that the case sample (ED and PDs) and the control sample (ED without PDs) were rather homogeneous. Differences were found in heteromasturbation, i.e., control cases were more concerned about masturbating their partners than ED + PDs cases, which can be explained by the fact that PDs are less empathic and more self-centred (Waldinger, 2015).

It can also be explained by the fact that control cases had higher scores on premature ejaculation, which may mean that people with PDs, who are characterised by being egosyntonic and not very empathic, could consider their intravaginal ejaculatory latency to be correct (Matesanz, 2006), despite having premature ejaculation, which does not occur in control cases more concerned with satisfying their partners.

Neuroticism has been associated with higher levels of sexual dissatisfaction and marital discomfort, suggesting a possible risk factor for the development and maintenance of male sexual problems (Gottman, 1994; Peixoto and Nobre, 2016), which corresponds with the data obtained in the research, as significant differences appear in the scales for anxiety and depressive neurosis, compulsivity and dependence PDs related to neuroticism. In the same sense, as mentioned, significant differences were found in the anxiety scale, as reflected in another study, Rosenheim and Neumann (1981), which demonstrated that men experiencing sexual difficulties presented more severe interpersonal anxiety, as found by other authors (Derogatis et al., 1981).

In the study, narcissistic individuals showed no significant differences compared to the control group, which is consistent with data from another study where it was found that narcissists improved better than other PDs on andrological treatment (Ajo et al., 2021). In the same way, histrionics also did better than other PDs in sexual therapy, which is possibly consistent with the findings of other authors (Bandini et al., 2009), who concluded that there is a positive relationship between sexual response and histrionic personality disorder in men. Finally, the data reflect a much better outcome with psychosexual therapy for the control group, which can be explained, in part, by the better couple relationship, which leads to greater collaboration in the therapeutic process. The control group maintained a better couple relationship, with significant differences compared to the ED and PDs group, which is consistent with the postulates of other authors who stated that people with PDs have difficulty in romantic relationships (Corral and Calvete, 2014; Kernberg, 2011) and obviously disorders of human significant relationships. (APA, 2024; Collazzoni et al., 2017). This has been specifically demonstrated in other studies with paranoid (Fisher et al., 2017), schizotypical (Jahangir et al., 2024; Okuda et al., 2015) and schizoid subjects (Brotto et al., 2010) who also commonly present sexual dysfunctions, personalities which in our study have a worse prognosis with respect to the control group without PDs.

In short, as other authors have concluded, the presence of PDs will have a negative influence on sexual response (Ciocca et al., 2023).

## 5 Conclusion

A very high percentage of men with psychogenic ED, who did not progress with IPDE5 treatment, had some PDs, 34% of whom met the criteria for more than one personality disorder, with compulsive personality predominating (76.92%), followed by dependent personality (35.90%) and schizoid personality (28.21%). Regarding the evolution of psychosexual therapy and its successful completion, there was a higher percentage of people who completed treatment in the control group compared to those in the case group (82.14% > 53.85%). Therefore, it can be concluded that ED comorbid with a PD has a worse therapeutic prognosis than ED without PD. This may be due, amongst other reasons, to the intrinsic characteristics of PDs and to the fact that men without PDs rated the quality of the couple’s relationship more positively than men with ED and PDs and, probably for this reason, the collaboration of the couple in the therapeutic process was greater. In fact, men without PDs masturbated their partners more to make them feel satisfied than men with PDs did.

In summary, men with psychogenic ED often have a personality disorder at the same time, with compulsive (rigid) personality predominating, and respond less well to psychosexual therapy than men with ED without a personality disorder.

The results of this study highlight the importance of systematically assessing the presence of personality disorders in men with psychogenic erectile dysfunction who do not respond to treatment with phosphodiesterase-5 inhibitors (PDE5). From a clinical perspective, this assessment may allow for more precise and personalised therapeutic interventions, which could significantly improve outcomes in these patients. In addition, the integration of therapeutic strategies targeting not only erectile dysfunction, but also the specific characteristics of the personality disorder, is suggested, with the aim of optimising the response to psychosexual treatment.

## 6 Strengths and limitations

The main strength of this work is that it was carried out with patients who came for consultation due to problems in their sexual function. This type of research allows us to adequately understand the needs of these patients and to improve the therapeutic system in order to offer quality, evidence-based care. In this sense it seems appropriate to point out the need to implement sexual psychotherapy with strategies to intervene, at the same time, on personality disorders.

However, one limitation is the need to confirm the results with a larger sample size.

## 7 Future research

Based on the results of this study, it would be of utmost relevance to investigate whether the application of strategies aimed at improving the most prevalent personalities comorbid with erectile dysfunction optimise erectile response to psychosexual treatment.

## Data Availability

The raw data supporting the conclusions of this article will be made available by the authors, without undue reservation.
